# Pure Bath Rooms and Clean Water Closets

**Published:** 1880-11

**Authors:** W. R. Monroe

**Affiliations:** Baltimore


### CORRESPONDENCE.

### PURE BATH ROOMS AND CLEAN WATER CLOSETS.

Having occupied a new dwelling house where an ordinary water closet occupied a place in the bath room on the second story end room, I was persuaded by a plumber to have a ventilator, size, two inch iron pipe, inserted, connecting the water closet inside, and penetrating through the wall, and extending on the outside upward above the roof of the house, and yet at times there would be an unpleasant odor in the bath room, arising from improper ventilation in the water closet. The flow of water, as in all ordinary closets, was from one orifice only, and in one direction, which frequently failed to cleanse the basin properly without extra attention.

Two years elapsed with this unsatisfactory arrangement, when at the suggestion of Messrs. E. S. Heath & Son, plumbers of this city, I agreed that they should remove the ordinary closet and put in "The Demarest" water closet. They also inserted a four inch ventilating pipe or chimney into the soil or waste pipe at its bend just outside the end wall, and extended it upward several feet above the wall of the house. The result has been most satisfactory. Now our bath room and water closet are entirely exempt from any unpleasant odor whatever, and are as comfortable and agreeable as any other room in our house. The "Demarest" has several advantages over other water closets. It has a deep oblong soil basin into which the water flows from two orifices in two opposite directions, which usually cleanses thoroughly in a few seconds as the plunge valve is raised.

The heavy plunge valve is then dropped and the basin soon fills and remains half full of pure water all the time or until used again. For personal comfort, cleanliness and health, we would recommend "The Demarest" to all families. We would not be without it for any reasonable amount of cost.

BALTIMORE, NOV., 1880. W. R. MONROE, M. D.

